# Supplementary material for: An integrated in silico immuno-genetic analytical platform provides insights into COVID-19 serological and vaccine targets
Source: Genome Med. 2021 Jan 7;13:4. doi: 10.1186/s13073-020-00822-6 (PMC7790334; doi:10.1186/s13073-020-00822-6)
Supplement: Supplementary file 1 — Additional file 1: Table S1. Extracted data from the Immuno-analytics tool. Mutations listed here have increased significantly over the 20-week period (weeks 20 to 40, year 2020) (see Fig. S2. for spike mutations A222V, S477N and L18F). Fig. S1. Screenshots from the Immuno-analytics webpage (http://genomics.lshtm.ac.uk/immuno). Fig. S2. Screen capture from ‘Mutation Tracker’ page tracing spike mutations accumulating in Europe, North America and Oceania since the last week of December 2019 (week 52) into 2020 (week 1 onwards). Mutations can be traced across continents by week on the ‘Mutation Tracker’ page. Mutations shown here are in the Spike (A222V, S477N and L18F). [file 13073_2020_822_MOESM1_ESM.pdf]

Table S1: Extracted data from the Immuno-analytics tool. Mutations listed here have increased significantly over the 20-week period (weeks 20 to 40, year 2020) (see **Fig. S2** for spike mutations A222V, S477N and L18F).

Protein Name	AA Position	Reference Allele	Alternative Alleles	Alternative Count
N	2	S	F:Y:P	3:7:20
N	220	A	V:T	11555:5
N	234	M	L:I	12:1681
N	376	A	V:T:S	2:1171:3
nsp12	176	A	V:S	121:1208
nsp12	767	V	E:L	1:1180
nsp13	218	K	R	1173
nsp13	261	E	K:D:G	1:1241:1
nsp3	1363	T	A:N:I	1:2:667
nsp3	1736	A	V	1057
nsp4	324	M	K:I:V	1:1244:3
nsp5	241	P	S:T:H:L	11:4:14:36
nsp8	95	L	F	109
orf10	30	V	A:I:L	2:2:11619
orf7a	65	F	S:L	1:20
orf7b	5	S	L:P:T	550:1:1
orf8	121	I	F:T:S:L:V	9:1:1:69:15
S	18	L	F:I	5888:1
S	222	A	T:V:P:I:S:F	4:11819:2:1:12:1
S	477	S	T:R:G:I:N:K	1:19:2:59:9811:1

(A)

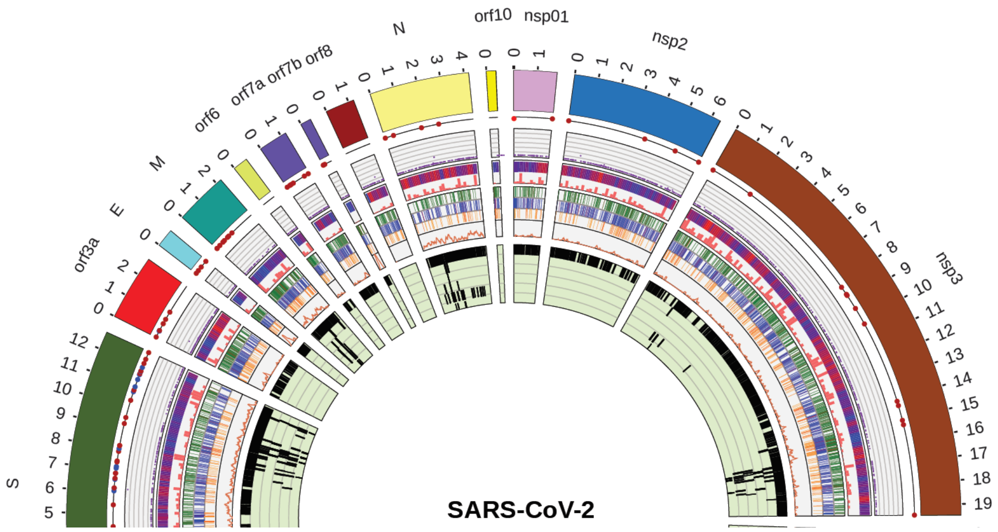

(B)

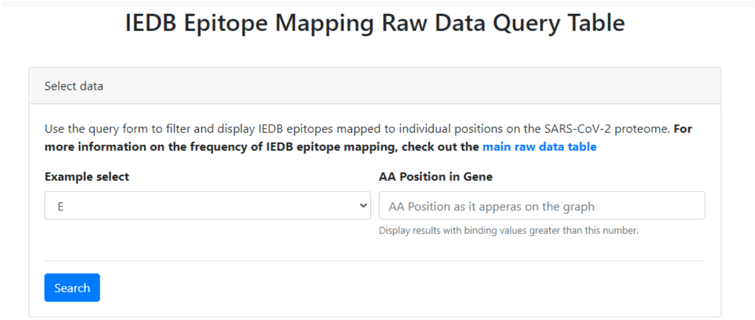

(C)

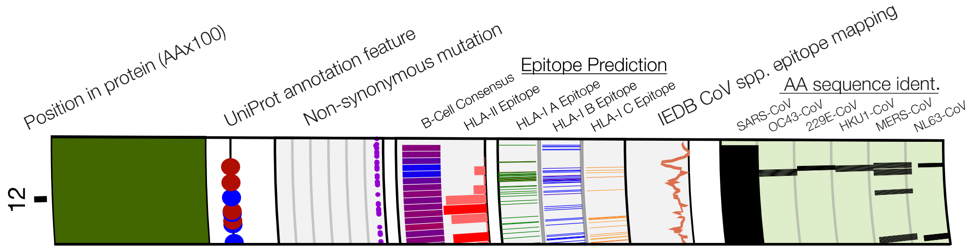

Fig S1. Screenshots from the Immuno-analytics webpage (http://genomics.lshtm.ac.uk/immuno). **(A)** Interactive plot view; **(B)** A search tool in a table format; **(C)** Integrated mutation, epitope and homology view

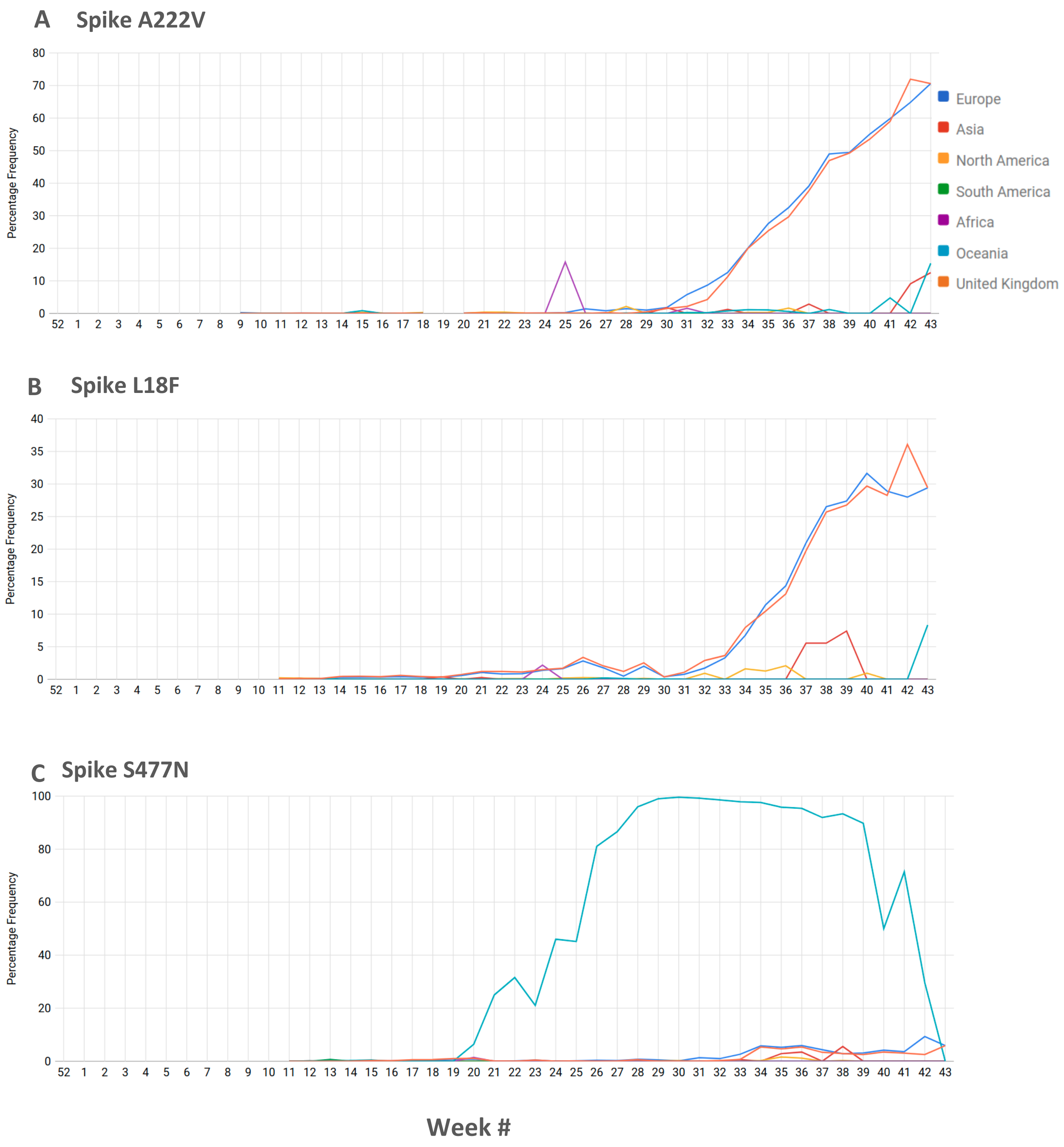

Fig S2. Screen capture from ‘Mutation Tracker’ page tracing spike mutations accumulating in Europe, North America and Oceania since the last week of December 2019 (week 52) into 2020 (week 1 onwards). Mutations can be traced across continents by week on the ‘Mutation Tracker’ page. Mutations shown here are in the Spike (A222V, S477N, and L18F).
